# Oviposition Behavior and Distribution of *Eucryptorrhynchus scrobiculatus* and *E. brandti* (Coleoptera: Curculionidae) on *Ailanthus altissima* (Mill.)

**DOI:** 10.3390/insects10090284

**Published:** 2019-09-04

**Authors:** Gan-Yu Zhang, Ying-Chao Ji, Peng Gao, Jun-Bao Wen

**Affiliations:** 1 Beijing Key Laboratory for Forest Pest Control, Beijing Forestry University, Beijing 100083, China; 2 College of Plant Protection, Shandong Agricultural University, Tai’an 271708, China

**Keywords:** *Eucryptorrhynchus scrobiculatus*, *Eucryptorrhynchus brandti*, *Ailanthus altissima*, oviposition behavior, oviposition sites, coexistence

## Abstract

(1) *Eucryptorrhynchus scrobiculatus* Motschulsky (Coleoptera: Curculionidae: Cryptorrhychinae) is a major quarantine forest pest in China. It often co-occurs with *E. brandti* (Coleoptera: Curculionidae: Cryptorrhychinae) on a single host *Ailanthus altissima* (Mill.) Swingle (tree of heaven). (2) In this study, to explain the coexistence of the two weevils on a single host, we investigated the oviposition behavior of *E. scrobiculatus* and oviposition sites of *E. scrobiculatus* and *E. brandti* under afield and laboratory conditions. (3) The characteristic behaviors of *E. scrobiculatus* females prior to oviposition included searching, locating, excavation, turning, locating the oviposition cavity, egg deposition, and hiding. (4) The oviposition sites used by *E. scrobiculatus* and *E. brandti* differed. *Eucryptorrhynchus scrobiculatus* females laid eggs in the soil near *A. altissima* and compound leaf petioles, while *E. brandti* females laid eggs in *A. altissima* trunks. The eggs in compound leaf petioles did not hatch in the field. (5) *Eucryptorrhynchus scrobiculatus* and *E. brandti* utilized different oviposition sites and these differences in habitat use may reduce the competition for resources between species during the larval period, thus facilitating their coexistence on *A. altissima*.

## 1. Introduction

*Ailanthus altissima* (Mill.) Swingle (Sapindales: Simaroubaceae), also called tree of heaven, is a species native to China and North Vietnam [1,2]. In China, it is widely distributed in 22 provinces. It is an important tree species in urban industrial and mining areas, because of its strong adaptability, low requirement for soil and high resistance to smoke and sulfur dioxide [3]. However, in America it is an invasive tree. In the 1700s *A. altissima* was first introduced into the United States and is now distributed, and has invaded, throughout North America, where it out competes with native vegetation [4,5,6,7].

*Eucryptorrhynchus scrobiculatus* Motschulsky (Coleoptera: Curculionidae: Cryptorrhychinae), which has strong host specificity, is a pest of *A. altissima* and its variant *A. altissima* var. Qiantouchun in China [8,9]. This weevil is native to China and is distributed in most areas where its host is found [10,11,12,13]. Larvae of *E. scrobiculatus* bore and feed on roots of *A. altissima*. It has one generation per year in China, with larval and adult overwintering [14,15]. After surviving hibernation, adult weevils crawl onto newly sprouted leaves and branches for nutritional supplementation. Gravid females lay eggs in the soil [16]. It takes about 15 minutes to lay one egg, and the average number of eggs per female is about 40 [15]. Usually, *E. scrobiculatus* is found with its close relative *E. brandti* (Harold) (Coleoptera: Curculionidae: Cryptorrhychinae), both of which are detrimental to *A. altissima* [9,17]. There are many similarities in the life history characteristics of the two species, such as they have one generation per year in China, with larval and adult overwintering; males and females can be mated multiple times; females begin oviposition about 30 days after mating; females lay one egg at a time, and so on [7,8,14,15,18]. However, the most obvious difference is that the larvae of *E. brandti* bore and feed on trunks of *A. altissima* [7,17]. In some areas of China, 80%–100% of *A. altissima* are attacked by *E. scrobiculatus* and *E. brandti*, causing 12%–37% mortality [17,19]. The particularity of *A. altissima* in China and the United States determines the difference in the status of these two weevils in these two regions. *Eucryptorrhynchus scrobiculatus* and *E. brandti* are major pests of *A. altissima* in China but are potential biological control agents in North America [2,7,17,19].

Species distributions across a landscape depend on conditions that define their fundamental ecological niche (comprised of all of the physical, chemical, and biological conditions required by a species for survival, growth, and reproduction), which is regarded as a multidimensional space of resources and conditions that allow the persistence of individuals at explicit sites [20]. For species that coexist, competition is probably the main interaction that reduces their realized niche (describing the part of the fundamental niche that a species actually occupies) [21,22]. In China, these two curculionid species are valuable for studies of ecological adaptability because they only harm a single host *A. altissima*, and coexist on this host [8,19,23]. It has reported that the genetic distance between *E. scrobiculatus* and *E. brandti* is 0.173, the differentiation time of the two weevils can be traced back to about 3.76 million years ago. It can be speculated that before this time, the two beetles may be the same species. In order to reduce intra-species competition to adapt to changes in certain environments, they form niche differentiation, groups occupying different niches have barriers to gene communication, and new species are formed through reproductive isolation [24]. In the long-term evolution process, *E. scrobiculatus* and *E. brandti* formed different survival strategies to achieve coexistence on the single host *A. altissima*. For example, from the perspective of adults, Ji [25] investigated the resource allocation of the *E. scrobiculatus* and *E. brandti* adults, including the activity time of a day, the activity range and feeding sites on the tree, and found that niche differentiation plays an important role in the coexistence of *E. scrobiculatus* and *E. brandti*. The importance of coexistence of *E. scrobiculatus* and *E. brandti* is followed by trophic niche differentiation, vertical spatial niche differentiation, and cardinal direction spatial niche differentiation. *Eucryptorrhynchus scrobiculatus* occurred more frequently on one-year-old branches, perennial branches, and petioles, and *E. brandti* occurred more on the stem. Furthermore, from the perspective of larvae, it has reported *E. scrobiculatus* larvae feed on the roots and *E. brandti* larvae feed on the trunks of *A. altissima* [26,27,28]. The differentiation of the feeding sites of larvae between the two weevils is also one of the reasons why they can coexist on the same host. However, the selection of female oviposition sites is closely related to the adaptability of offspring [29], especially when larvae cannot move to different oviposition positions. The larvae of the two species have a very small range of activity, therefore, the oviposition sites of adults may determine the place where larvae develop. Therefore, we hypothesize that *E. scrobiculatus* and *E. brandti* utilized different oviposition sites and these differences in habitat may reduce the competition for resources between species during the larval period, thus facilitating their coexistence on *A. altissima*. To test this hypothesis, we studied the oviposition behavior of *E. brandti* females. The oviposition process of *E. brandti* females consists of nine main sequential steps: Wide-area search, site location, oviposition cavity excavation, excitation, turning, locating the oviposition cavity, egg deposition, egg concealment, and resting [18]. However, the precise oviposition behavior of *E. scrobiculatus* is unclear, although brief descriptions have been reported [14,15,16]. In this study, our objectives were: (1) To provide a detailed description of the oviposition behavior of *E. scrobiculatus* and compare these behaviors with those of *E. brandti*, (2) to determine the oviposition sites and distributions of *E. scrobiculatus* and *E. brandti* in the field, and (3) to discuss possible relationship between oviposition and coexistence of the two weevils.

## 2. Materials and Methods

### 2.1. Study Area

The study was carried out at two spots in Ningxia Hui Autonomous Region, Lingwu farm (38°07′7.76″N, 106°18′32.19″E) and Xiaoxingdun Village (38°51′27.44″N, 106°31′33.03″E). An *A. altissima* forest was located along a ditch on both sides of farmland. *Eucryptorrhynchus scrobiculatus* and *E. brandti* occurred together on *A. altissima*.

### 2.2. Observation of the Oviposition Behavior of E. scrobiculatus

A total of 80 pairs of *E. scrobiculatus* adults were tracked in an *A. altissima* forest under natural conditions in the field (each pair consisted of a female and a male). The oviposition process of *E. scrobiculatus* was filmed using a digital camera (PowerShot G7 X Mark II; Canon, Beijing, China). To minimize disturbance to the gravid females, the camera was mounted on a tripod (NB-238, height range: 300–1510 mm).

In the lab, 2–3 *A. altissima* seedling sprouts in good condition were planted in a plastic pot (diameter 65 cm, height 50 cm), which was filled with soil collected from the vicinity of *A. altissima*. Two pots were covered with a pre-set white domed net (150 × 200 × 170 cm), and five pairs of adult *E. scrobiculatus* which showed active oviposition behavior (laying eggs) were selected and placed in the net from 30 pairs, one pair of *E. scrobiculatus* observed at a time, and 24 hours for each pair. The oviposition behavior of *E. scrobiculatus* was filmed using a camera mounted on a tripod also. For subsequent behavior comparison, the light condition (the net was illuminated from 08:00 to 20:00 by an LED fluorescent lamp) and methods were the same as we had used for *E. brandti* adults [18].

### 2.3. Investigation of Oviposition Sites of E. scrobiculatus and E. brandti in the Field

During the peak period of oviposition (mid-May to late June in Ningxia Hui Autonomous Region), an *A. altissima* tree was randomly selected with at least five pairs of *E. scrobiculatus* adults and *E. brandti* adults (each pair consisted of a female and a male). Insertion of the rostrum into the oviposition substrate during the peak period of oviposition was considered a potential oviposition behavior. Females were tracked until eggs were deposited and oviposition sites were recorded. The horizontal and vertical distances of the oviposition site from the *A. altissima* trunk were measured using a tape measure and an altimeter. One tree was observed from 9:00–17:00 every day, and 15 trees were surveyed.

These trees of heaven are tall (the height is about 7–12 meters) and leafy in May–July in the Ningxia area, and it is difficult to observe whether weevils on the branches are laying eggs. A ladder was used to climb the trees and find unknown eggs in compound leaf petioles of *A. altissima*. Forty trees were randomly selected to search for nine compound leaf petioles with unknown eggs. After recording the horizontal and vertical positions, eggs were collected from the branches. 

The eggs were taken to the laboratory for cleaning and incubation in Petri dishes. These dishes were supplemented with water-soaked absorbent cotton at 25°C, 48% r.h., and a 16L: 8D photoperiod in the laboratory. When the unknown eggs hatched, larvae were placed in a 5 mL freezing tube filled with anhydrous ethanol and stored in a refrigerator at −20 °C. The larvae of *E. scrobiculatus* (collected from the soil near *A. altissima*) and larvae of *E. brandti* (collected from the trunk of *A. altissima*) acquired directly in the field were stored in the same way. In this study, two *E. scrobiculatus* larvae, two *E. brandti* larvae, and nine larvae hatched from unknown eggs were randomly selected for classification and identification by PCR. The TIANamp Genomic DNA Kit (DP304; Tiangen, Beijing, China) was used to extract DNA. A 631 bp gene fragment was amplified using the degenerate primer pair 5′-GGWATAGATGTWGAYACWCGTGCTTA-3′ and 3′-TTCCACCWGCAGATCATAGTTATG-5′. PCR was carried out according to the instructions provided with the Fast HiFidelity PCR Kit (KP202, Tiangen, Beijing, China).

In addition, during the investigation, we found that there were some dead eggs on petioles. We suspect that the eggs laid on petioles are unlikely to hatch into larvae in the wild. To test this, 15 trees were randomly selected, and two compound leaf petioles of each tree were randomly selected and covered with a net (100 mesh, 45 × 40 cm). Five pairs of adults were put inside the net, allowed to lay eggs, based on the presence of oviposition scars on the petioles (see Figure S1A,B), and then removed. Petioles were kept covered with the net to prevent hatching larvae from falling. We observed and counted the hatching of eggs every day for a total of 15 days.

### 2.4. Oviposition Preference of E. scrobiculatus and E. brandti in the Laboratory

Trunks (3 cm in diameter) of five healthy *A. altissima* trees were cut into 7 cm-long pieces. Compound leaf petioles of 0.5 cm in diameter from healthy *A. altissima* were removed from the leaves and cut into 3 cm-long pieces. The soil was collected near *A. altissima* with 10%–20% humidity (the soil water content was measured with a soil hygrometer, Beikeshi, Weifang, China). These soil samples were placed at the bottom of 30 plastic boxes (21 cm × 13 cm × 13 cm) at a height of 1 cm. One trunk bolt, one compound leaf petiole, and five pairs of *E. scrobiculatus* adults and *E. brandti* adults (provided supplemental nutrition 7–10 days after emergence) were placed into each box. Eggs on each part were counted every other day until no eggs were found in all boxes. Fresh materials were changed and the soil was watered to maintain moisture. The experiments were performed in the lab under natural conditions to simulate the natural conditions in the field. The temperature was from 16.8 °C to 37 °C, the humidity from 19.4%–91.2%, and the dew point temperature from −5.5 °C to 27.7 °C.

### 2.5. Data Analyses

Films of oviposition behavior were analyzed using The Observer XT 14 (Noldus, Wageningen, Netherlands). Data were subjected to statistical analysis using SPSS 11.0 for Windows (SPSS, Chicago, IL, USA). One-way analysis of variance (ANOVA) was used to compare the time cost of different behaviors and the mean numbers of eggs for different substrates in the oviposition preference tests. MEGA 7.0 software (https://www.megasoftware.net/) and Figtree 1.4.3 (http://tree.bio.ed.ac.uk/software/figtree/) were used to analyze the gene sequences for the larvae that hatched from unknown eggs and the larvae of *E. scrobiculatus* and *E. brandti*.

## 3. Results

### 3.1. Behavior of E. scrobiculatus Females during Oviposition

**Figure 1:**
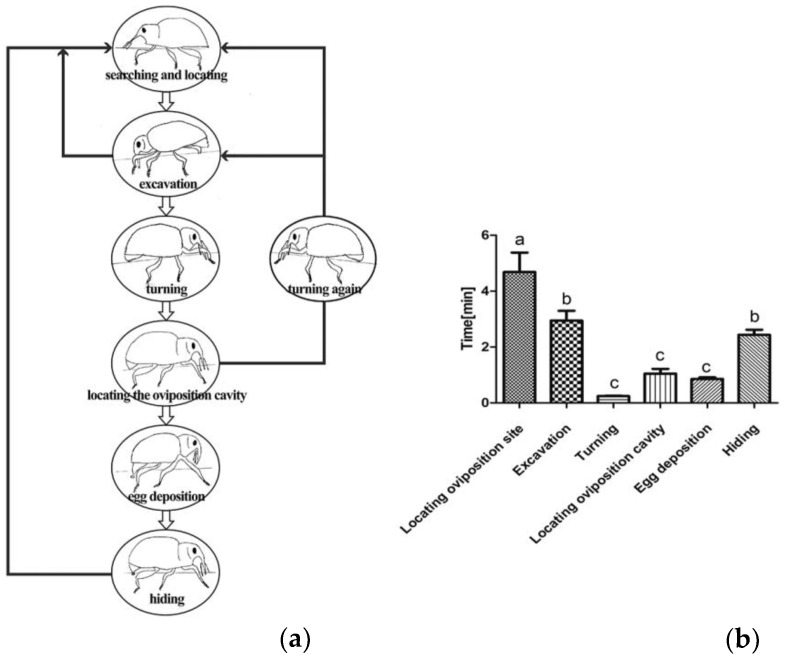
Oviposition performance of *Eucryptorrhynchus scrobiculatus* females. (**a**) Representative diagram of sequential pre- and post-oviposition behavior displayed by gravid females *Eucryptorrhynchus scrobiculatus*; (**b**) duration of steps in the oviposition sequence of *Eucryptorrhynchus scrobiculatus* (mean ± SE, N = 32). The level of significance was set at *p* < 0.001.

**Gravid:***Eucryptorrhynchus scrobiculatus* females displayed several prominent behaviors for oviposition. These included (1) searching and locating, (2) excavation, (3) turning, (4) locating the oviposition cavity, (5) egg deposition, and (6) hiding. A description of these behaviors is provided in Figure 1.

**Searching and locating:** Before oviposition, gravid females searched for a suitable oviposition location. In this process, females walked on the land around *A. altissima* and hit the surface of the soil with their antennae to detect oviposition sites. When a position was initially detected, they stopped walking and inserted their rostrum into the soil to locate the site, this behavior lasted for 4.69 ± 0.69 min (mean ± SE) (Figure 1).

**Excavation:** The female moved forward, shifted its weight onto the rostrum, and inserted the rostrum deeper into the soil. An oval oviposition cavity was slowly produced. In the drilling process, the female continued to use its antennae for detection; if the site was not suitable to lay eggs, the individual gave up immediately and searched for a new oviposition site. Oviposition cavity excavation lasted for 2.94 ± 0.35 min (Figure 1).

**Turning:** When the entire rostrum of *E. scrobiculatus* females was inserted into the soil and the abdomen began to bend, excavation was nearly complete. Females then turned their bodies 180 degrees for oviposition. This behavior took approximately 0.24 ± 0.02 min (Figure 1).

**Locating the oviposition cavity:** After turning their bodies, females moved their abdomens to look for oviposition cavities with the extension of the ovipositor. This stage was a crucial determinant of the success of oviposition. One female could deposit one egg if the ovipositor was successfully inserted into the oviposition cavity; otherwise, the female would abandon the oviposition cavity and start from the beginning (i.e., searching and locating). Locating the oviposition cavity lasted for 1.05 ± 0.17 min (Figure 1).

**Egg deposition:** When locating the oviposition cavity, the female ejected a single egg from the ovipositor, accompanied by abdominal contraction and bending. This behavior lasted for 0.86 ± 0.06 min (Figure 1).

**Hiding:** After oviposition, females did not leave immediately. Instead, they used the abdomen to accumulate soil to cover the cavity, thereby protecting the newly produced egg. The process took approximately 2.44 ± 0.18 min (Figure 1).

### 3.2. Comparison of the Oviposition Behavior of E. scrobiculatus and E. brandti

**Figure 2:**
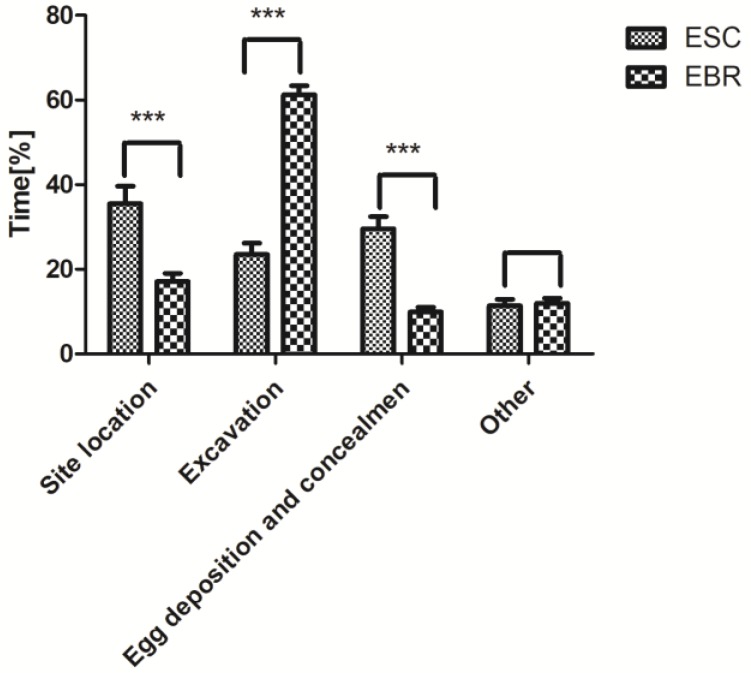
The percentages of time spent on some characteristic behaviors (characteristic behavior/total time) of *Eucryptorrhynchus scrobiculatus* (ESC) and *E. brandti* (EBR) (mean ± SE). The brackets represent a significant difference by the chi-square test (*** *p* < 0.001).

When the oviposition cavity was successfully excavated, the females of *E. brandti* performed a unique behavior in which they use the front tarsi to make an excitatory gesture to indicate excavation success [18], while *E. scrobiculatus* did not perform this behavior. In addition to this excitatory behavior, the two species exhibited similar oviposition behavior. *Eucryptorrhynchus scrobiculatus* and *E. brandti* females all used their rostrums to drill an oviposition cavity at the oviposition substrate and then turned their bodies to lay eggs. However, during the entire oviposition process, the time spent in the similar behavior between the two weevils was different. Based on previous studies on the oviposition of *E. brandti* [18], we compared the percentages of time spent on some behaviors between the two species. The results show that *E. scrobiculatus* spent more time on locating oviposition sites (*F* = 21.223, *p* < 0.001), and depositing and hiding eggs (*F* = 56.053, *p* < 0.001), but *E. brandti* spent more time on excavating (*F* = 120.002, *p* < 0.001) (Figure 2).

### 3.3. Eucryptorrhynchus scrobiculatus Lay Eggs in Compound Leaf Petioles

**Figure 3:**
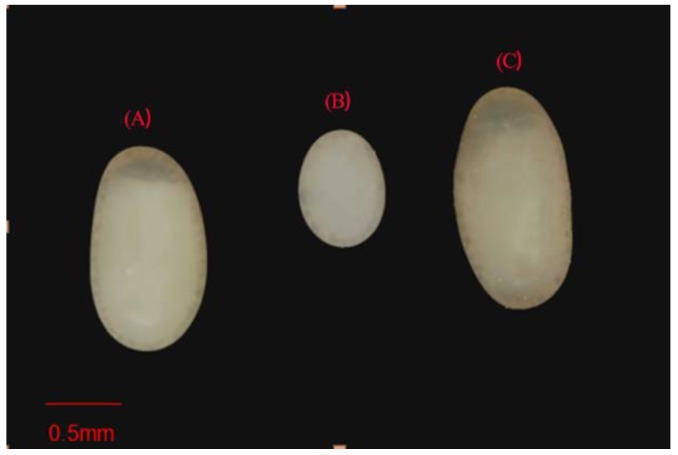
Morphological comparison of eggs. (**A**) *Eucryptorrhynchus scrobiculatus* egg, (**B**) *E. brandti* egg, and (**C**) unknown egg.

*Eucryptorrhynchus scrobiculatus* eggs are about 1.7 mm long and 0.9 mm wide. In *E. brandti*, the egg length is about 1.0 mm and the egg width is about 0.8 mm [26]. The unknown eggs were similar in size to those of *E. scrobiculatus*, with a length and width of about 1.77 ± 0.08 mm and 0.88 ± 0.05 mm, respectively (Figure 3).

**Figure 4:**
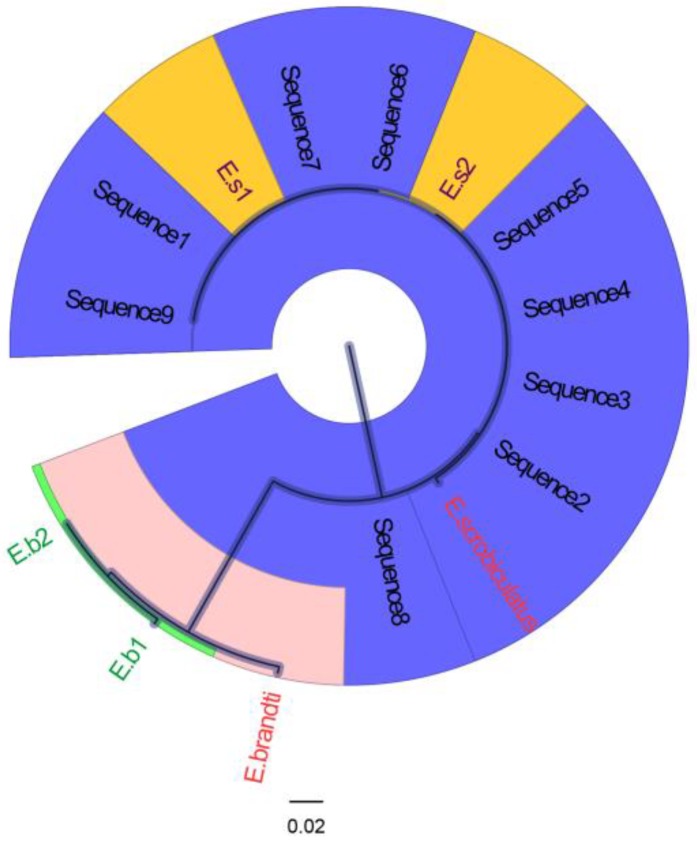
The phylogenetic tree of DNA sequences. *Eucryptorrhynchus scrobiculatus* and *E. brandti*, marked in red, are the mitochondrial sequences obtained from NCBI. E.b1 and E.b2, marked in green, indicate the DNA sequences of *E. brandti* larvae collected from the trunk of *A. altissima*. E.s1 and E.s2, marked in yellow, indicate the DNA sequences of *E. scrobiculatus* larvae collected from the soil near *Ailanthus altissima*. Sequences 1–9 are the DNA sequences of larvae hatched from unknown eggs.

Gene sequences of larval DNA were compared with the mitochondrial genomes (COI) of *E. scrobiculatus* (KP410324) and *E. brandti* (KP455482) obtained from NCBI. As shown in Figure 4, the sequences obtained from larvae formed two distinct clusters, and *E. scrobiculatus* and *E. brandti* each occupied a cluster. The gene sequences of nine larvae that hatched from unknown eggs were assigned to the same cluster as the *E. scrobiculatus* sequences. This result suggests that the unknown eggs were deposited by *E. scrobiculatus*, indicating that *E. scrobiculatus* females can lay eggs in compound leaf petioles.

After the *E. scrobiculatus* females lay eggs in the petiole of the compound leaf, we observed for 15 consecutive days, and no larvae were found near the oviposition cavity or in the net. A total of 27 dried eggs were found after peeling off the petioles of the compound leaves (Figure S1C,D). This indicates that the eggs in the petiole are very unlikely to hatch into larvae in the field.

### 3.4. Oviposition Sites Distribution in the Field

**Figure 5:**
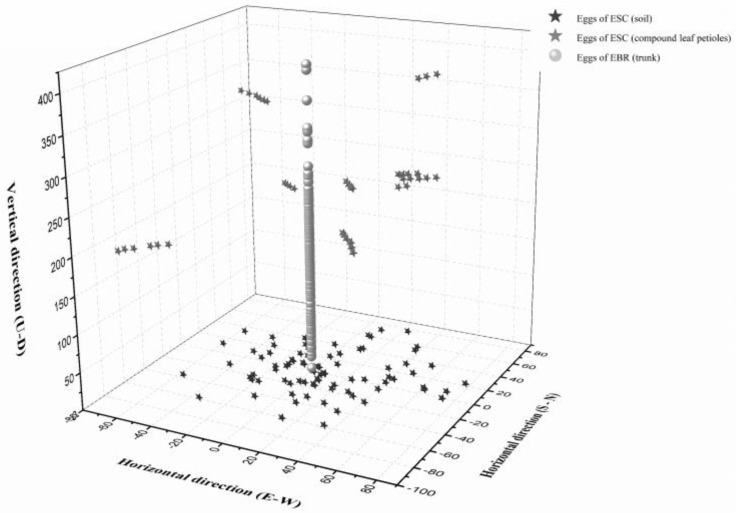
Oviposition site distributions for *Eucryptorrhynchus scrobiculatus* (ESC) and *E. brandti* (EBR) centered on the trunk of *Ailanthus altissima*. The length is measured in centimeters.

In the *A. altissima* forest, we investigated 131 oviposition sites of *E. scrobiculatus*, of which 91 were in the soil near *A. altissima* and 40 were in the compound leaf petioles of *A. altissima*. We also investigated 196 oviposition sites of *E. brandti*, all of which were found under the bark of *A. altissima* trunks. We detected obvious differentiation between the oviposition sites of *E. scrobiculatus* and *E. brandti* (Figure 5). *Eucryptorrhynchus scrobiculatus* females laid eggs in the soil near *A. altissima* and compound leaf petioles, while *E. brandti* females laid eggs in *A. altissima* trunks. The oviposition sites of *E. scrobiculatus* were mainly concentrated in the soil 0–40 cm in the horizontal direction from *A. altissima*, with a maximum distance of about 77 cm from *A. altissima*. Eggs laid in compound leaf petioles did not exist alone. Generally, 3–8 eggs can be found on a compound leaf petiole. *E. brandti* laid eggs at any height on the tree trunk ranging from 5 cm to 405 cm.

### 3.5. Oviposition Preference of E. scrobiculatus and E. brandti in the Laboratory

**Figure 6:**
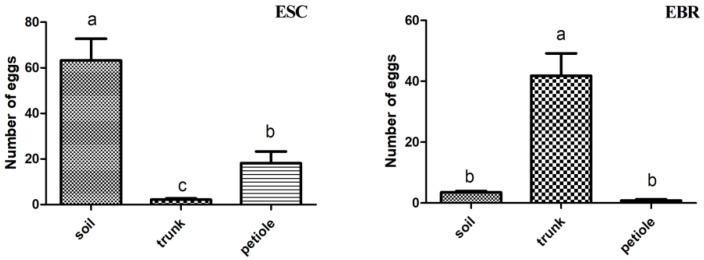
Number of eggs in oviposition preference tests. The y-axis shows average number of eggs per substrate laid by *Eucryptorrhynchus scrobiculatus* (ESC, **left panel**) and *E. brandti* (EBR, **right panel**). The *x*-axis indicates the test materials (mean ± SE, N = 30). The brackets represent a significant difference by the chi-square test (*** *p* < 0.001, different letters “a” “b” “c” indicate statistically significant differences).

In the laboratory (Figure 6), *E. scrobiculatus* females preferred to lay eggs in the soil, followed by petioles, and rarely in trunks (*F* = 26.212, *p* < 0.001); *E. brandti* females laid eggs mainly in tree trunks and rarely in soil and petioles (*F* = 29.072, *p* < 0.001). We found that although *E. scrobiculatus* females lay eggs in the trunk bolts in laboratory oviposition tests, they only lay eggs in the phloem of the cross-section of the trunk and rarely in the phloem under the bark of the trunk, while *E. brandti* females not only laid eggs in the phloem of the cross-section of the trunk but also in the phloem under the bark it has bitten.

## 4. Discussion

Egg-laying behavior varies among insect groups, but all taxa show similar basic procedures, including wide-range host search and orientation, close positioning and settlement, host identification and selection (accept or reject), and egg deposition [30]. Determining the specific oviposition sequence of *E. scrobiculatus* requires careful observation. Gravid females first probe the soil surface with their antennae and mouthparts, which is typical of Curculionoidea, and has been observed in other species as well [31]. The whole oviposition process for *E. scrobiculatus* was quite similar to that of *E. brandti* [18]. Both species use their rostrums to drill an oviposition cavity at the oviposition substrate and then turn their bodies to lay eggs. However, during the entire oviposition process, *E. brandti* spent most time on excavating while *E. scrobiculatus* spent most time on locating oviposition sites (Figure 1 and Figure 2). When the oviposition cavity is successfully excavated, the females of *E. brandti* perform a unique behavior in which they form an excitatory gesture with the front tarsi to indicate excavation success. However, *E. scrobiculatus* does not perform this behavior.

This is the first report of *E. scrobiculatus* oviposition in compound leaf petioles. However, the eggs in compound leaf petioles cannot hatch in nature, although collected eggs hatched in dishes. The reasons for this phenomenon are unclear. The dynamics of biological evolution lie in mating, reproduction, and parenting [32]. However, it is not clear why egg laying in the petiole in *E. scrobiculatus* has not been eliminated during evolution or whether this behavior is ineffective. These issues require further study.

*Eucryptorrhynchus scrobiculatus* and *E. brandti* are trunk-boring pests and are highly host-specific to *A. altissima* [7]. Larvae cause the majority of damage in both weevils. Previous studies have shown that *E. scrobiculatus* larvae feed on the roots and *E. brandti* larvae feed on the trunk [26,27,28]. Therefore, the distribution and utilization of the same host resources by the two weevils at the larval stage plays an important role in their coexistence. This study results showed there was obvious differentiation in oviposition sites between *E. scrobiculatus* and *E. brandti*. *Eucryptorrhynchus scrobiculatus* laid eggs in the soil and compound leaf petioles of *A. altissima,* the eggs in compound leaf petioles did not hatch in the field, while *E. brandti* laid eggs in the trunks of *A. altissima*. Thus, the difference in feeding sites between the larvae of *E. scrobiculatus* and *E. brandti* is caused by the difference in oviposition sites between the females of *E. scrobiculatus* and *E. brandti.* The soil nearby *A. altissima* is the feeding and activity sites of *E. scrobiculatus* larvae and the trunk of *A. altissima* is the feeding and activity sites of *E. brandti.* This result supports the hypothesis that *E. scrobiculatus* and *E. brandti* utilized different oviposition sites and these differences in habitat use may reduce the competition for resources between species during the larval period, thus facilitating their coexistence on *A. altissima*.

In this study, the laboratory tests of oviposition preferences of these two weevils was consistent with our field observations. The difference is that we found *E. scrobiculatus* female lay eggs in the trunk bolts in laboratory oviposition tests. However, even if they lay eggs in the trunk bolts, they only lay eggs in the phloem of the cross-section of the trunk and rarely in the phloem under the bark of the trunk, while *E. brandti* females not only laid eggs in the cross-section of the trunk bolts but also under the bark it has bitten. Oviposition behavior has shown that both types of weevils needed to use their rostrum to bore an oviposition cavity before laying eggs. Therefore, we speculate that the bark is too hard for *E. scrobiculatus* females to drill cavities. In addition, their eggs are larger than those of *E. brandti* (Figure 3), and the time of oviposition has shown that *E. brandti*, which has smaller eggs, spent a large amount of time in excavating a cavity. If *E. scrobiculatus* lays eggs in bark it needs to consume more time and energy in the preparation of the oviposition cavity. Thus, *E. scrobiculatus* lays eggs in the soil and petioles in which it is relatively easy to make cavities. In this study, we did not investigate the hatching and development of larvae in different oviposition substrates. It was necessary to remove the eggs in the process of quantifying oviposition, thus altering the original state of the deposited eggs.

In both *E. scrobiculatus* and *E. brandti*, an important step for oviposition site preparation is the excavation of an oviposition cavity using the mouthparts at the top of the rostrum [15,16,33]. Oviposition site preparation is considered the key adaptation facilitating the circumvention of the physical defenses of the plant (shells and spines), avoidance of the desiccation of the larvae, and initiation and maintenance of attachment to the host [34]. The wide range of host plants attacked by species in the genus *Curculio* is hypothesized to be a result of ecomorphological adaptations to oviposition site, and seed size has been postulated to be responsible for morphological changes in rostrum size [35]. A recent series of studies show in great detail that across different populations of the weevil species *Curculio camelliae* (Curculionidae), the relative female rostrum length is strongly correlated with the relative thickness of the pericarp of the fruits of its host plant, *Camellia japonica* [36,37,38]. Therefore, we speculated that the difference in sites for egg laying between *E. scrobiculatus* and *E. brandti* may be caused by the adaptation of their rostrums to the ecological properties of the oviposition sites, and the morphology of their rostrums may be related to the hardness of the oviposition substrate surface. However, the choice of insect oviposition sites is inherently a complicated process, which may be related to the volatiles, color, nutritional status of the oviposition sites and offspring adaptability, etc., or related to the interaction of multiple factors. The reasons for the different oviposition sites of these two species of weevils require further study [39,40,41].

## 5. Conclusions

In this study, we described the main oviposition behaviors in detail and further recorded the specific behaviors in the form of hand-drawn diagrams. The characteristic behaviors of *E. scrobiculatus* females prior to oviposition included searching, locating, excavation, turning, locating the oviposition cavity, egg deposition, and hiding. In addition, we found there were obvious differentiation in oviposition sites between *E. scrobiculatus* and *E. brandti* in the field. *E. scrobiculatus* females laid eggs in the soil near *A. altissima* and compound leaf petioles, while *E. brandti* females laid eggs in *A. altissima* trunk, and the eggs in compound leaf petioles did not hatch in the field. The different egg laying position of the two species females allow the larvae to feed on different areas, reducing the competition between them for the single host *A. altissima.* Therefore, this oviposition strategy of females contributes to the coexistence of the two species on a single host *A. altissima*. Based on preference test and behavioral comparison, we speculated the difference in oviposition sites of the two weevils might be related to the structural adaptability of rostrum to oviposition substrate, this is what we will focus on in the future.

## Supplementary Materials

The following are available online at https://www.mdpi.com/2075-4450/10/9/284/s1; **Figure S1.** Compound leaf petioles after oviposition by *Eucryptorrhynchus scrobiculatus* females. (**A**) Oviposition cavity in the compound leaf petiole within two days of laying eggs; (**B**) eggs in the compound leaf petiole within two days of laying eggs; (C) oviposition cavity in the compound leaf petiole 15 days after laying eggs; (**D**) eggs in the compound leaf petiole in the compound leaf petiole 15 days after laying eggs. **Figure S2.** Egg laying figures of two species of weevils. (**a**) *Eucryptorrhynchus scrobiculatus* is laying its egg in the soil; (**b**) *Eucryptorrhynchus brandti* is laying its eggs in the trunk.
