# Supplementary material for: Oviposition Behavior and Distribution of Eucryptorrhynchus scrobiculatus and E. brandti (Coleoptera: Curculionidae) on Ailanthus altissima (Mill.)
Source: Insects. 2019 Sep 4;10(9):284. doi: 10.3390/insects10090284 (PMC6780713; doi:10.3390/insects10090284)
Supplement: Supplementary file 1 [file insects-10-00284-s001.zip › insects-562596/insects-562596-s.docx]

**Supplementary Materials:**

**
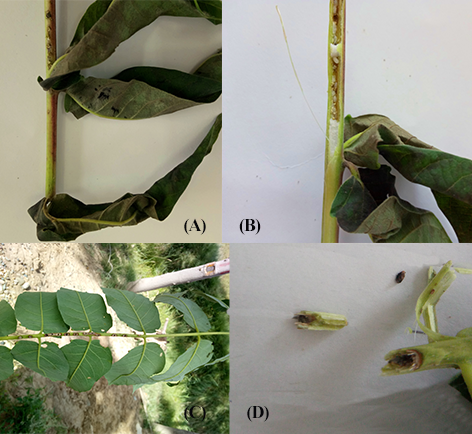
**

**Figure S1.** Compound leaf petioles after oviposited by *Eucryptorrhynchus scrobiculatus* females. (A) Oviposition cavity in the compound leaf petiole within 2 days of laying eggs; (B) Eggs in the compound leaf petiole within 2 days of laying eggs; (C) Oviposition cavity in the compound leaf petiole 15 days after laying eggs; (D) Eggs in the compound leaf petiole in the compound leaf petiole 15 days after laying eggs.


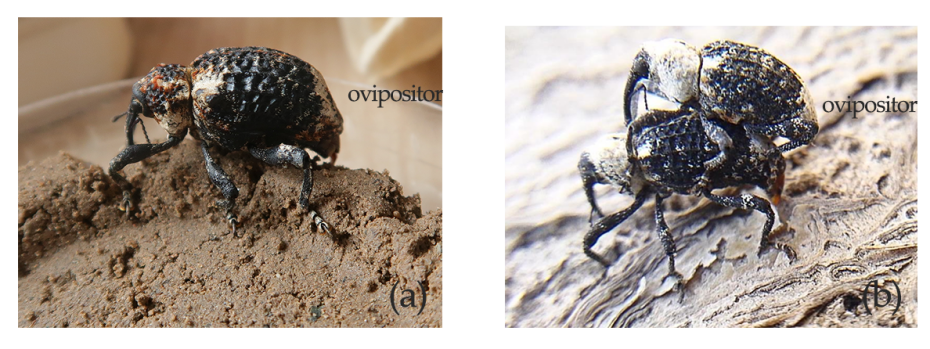


**Figure S2**. Egg laying figures of two species of weevils. (a) *Eucryptorrhynchus scrobiculatus* is laying its egg in the soil; (b) *Eucryptorrhynchus brandti* is laying its eggs in the trunk
